# An evaluation of the real world use and clinical utility of the Cxbladder Monitor assay in the follow-up of patients previously treated for bladder cancer

**DOI:** 10.1186/s12894-020-0583-0

**Published:** 2020-02-11

**Authors:** Madhusudan Koya, Sue Osborne, Christophe Chemaslé, Sima Porten, Anne Schuckman, Andrew Kennedy-Smith

**Affiliations:** 1 Waitemata District Health Board, Auckland, New Zealand; 2 Mid Central District Health Board, Palmerston North, New Zealand; 3 University of California San Francisco, San Francisco, CA USA; 4 USC Institute of Urology, USC/Norris Comprehensive Cancer Center, University of Southern California, Los Angeles, CA USA; 5 Capital & Coast District Health Board, Wellington, New Zealand

**Keywords:** Bladder cancer, Cystoscopy, Recurrence, Genomic rule-out test

## Abstract

**Background:**

Surveilling recurrent urothelial carcinoma (UC) requires frequent cystoscopy, which is invasive, expensive and time-consuming. An accurate urinary biomarker has the potential to reduce the number of cystoscopies required during post-treatment surveillance.

**Objective:**

To audit the clinical utility of a new surveillance protocol incorporating the Cxbladder Monitor (CxbM) test in real-world practice.

**Methods:**

Three hospitals implemented a new surveillance protocol. Patients were risk stratified, and then provided urine samples for CxbM testing. Low-risk CxbM-positive patients and all high-risk patients had cystoscopy at 2–3 months. Low-risk CxbM-negative patients had cystoscopy at ~ 12 months.

**Results:**

443 CxbM tests were conducted on samples from 309 patients: 257 (83.2%) low-risk and 52 (16.8%) high-risk. No pathology-confirmed recurrences were seen in low-risk CxbM-negative patients (*n* = 108) during the first post-CxbM cystoscopy undertaken a mean ± SD 10.3 ± 3.9 months after testing. Three recurrences were detected during cystoscopy at 2.7 ± 3.4 months in 53 low-risk CxbM-positive patients. In 49 high-risk patients, 39 (79.6%) were CxbM-negative with no pathology-confirmed recurrences. Ten high-risk patients (20.4%) were CxbM-positive with four confirmed recurrences; 2 high-grade and 2 low-grade. The median time to first cystoscopy was 12.13 (95% CI: 11.97–12.4) months in patients with a CxbM-negative result versus 1.63 (95% CI: 1.13–2.3) months in patients with a CxbM-positive result (*p* < 0.00001). No positive cases were missed, no patients progressed to invasive or metastatic disease, and no patient died of cancer over 35 months of follow-up.

**Conclusions:**

CxbM accurately identified a high proportion of patients (77.8%) who were safely managed with only one cystoscopy per year. Including CxbM in the protocol for patient surveillance provided clinical utility by reducing the average number of annual cystoscopies by approximately 39%, thereby sparing patients the potential discomfort and anxiety, without compromising detection rates. No advantage was observed for risk stratification prior to CxbM.

## Background

Most forms of urothelial carcinoma (UC) are cancers of the bladder. Although most cases of non-muscle-invasive UC can be treated, bladder cancer has a high rate of recurrence. Even patients with low-grade or low-risk UC require regular surveillance after treatment [1]. As a result, bladder cancer carries the highest per-lifetime, per-patient cost of any type of cancer [2], with 60% of the total cost attributable to surveillance and recurrence [3].

Major guidelines recommend risk-adjusted surveillance or active surveillance strategies for patients after treatment for UC, cystoscopy, cytology and imaging for diagnosis and monitoring in most cases [4–10]. The first cystoscopy should be at 3–4 months after the completion of treatment [10]. If this is negative, patients should undergo cystoscopies over longer time periods for low-risk vs intermediate vs high-risk categorizations [7, 10]. All patients with a recurrence start their evaluation sequence again. Costs rapidly accrue because cystoscopy is an invasive endoscopic procedure requiring local anesthesia, expensive equipment and expertise. Patients often find the procedure disagreeable and time-consuming, keeping them from work and life activities. Reluctance to undergo cystoscopy impacts patient compliance with guideline-recommended surveillance protocols [11], which may increase disease progression.

There is evidence to suggest that the benefit: risk equation for diagnostic and surveillance procedures does not necessarily favor current practices [12–14]. For example, American Urological Association (AUA) guidelines for the evaluation of hematuria recommend extensive and intensive use of tests and procedures, including CT imaging [10], impacting significantly on costs, compared with guidelines that recommend less intensive assessment [12]. Comparatively less intensive approaches miss more low-grade UCs, but with fewer adverse outcomes [12]. Moreover, studies specifically investigating the surveillance of patients after treatment for UC suggest that low-risk patients often undergo more frequent surveillance cystoscopies than is recommended by AUA guidelines [13, 14]. Such overuse is associated with increases in surgical procedures and total medical costs, without reducing risk of UC progression or death [14].

The level of diagnostic performance of the current generation of urinary biomarker tests means that they now successfully reduce the need for invasive and expensive cystoscopy assessments in patients being managed for bladder cancer. Recent real-world evidence has been published investigating a new protocol that combines imaging with Cxbladder Triage™, an algorithm combining urinary biomarker data with patient phenotypic data, for hematuria patients being evaluated for UC [15]. With the high negative predictive value (NPV) and high sensitivity of Cxbladder Triage, the new protocol provided a rule-out strategy that was able to safely identify patients without disease and avoid the need for cystoscopy in 32% of patients undergoing evaluation of hematuria [15].

The Cxbladder-Monitor (CxbM) test uses a similar ‘rule-out strategy’ to rule out the presence of UC among patients being evaluated for UC recurrence. CxbM quantifies urine mRNA levels of five cancer biomarkers [16, 17], and incorporates this information into a mathematical algorithm with clinical variables (primary versus recurrent UC and time since previous tumor resection) to derive a score with a binary outcome [16] (see Additional file 1). Prospective studies in patients undergoing surveillance for recurrent bladder cancer have reported sensitivity of between 91 to 95% for CxbM, and an NPV of 96 to 97% [16, 17].

Based on these published data, several of New Zealand’s public healthcare providers (PHP) have integrated CxbM into their routine clinical surveillance of patients for recurrence of bladder cancer. The new clinical practice alternates the use of CxbM and cystoscopy during regular surveillance of low-risk patients.

This real-world audit describes the use and outcomes of cystoscopy at these PHPs over a 35-month period after the inclusion of CxbM into the surveillance protocol, and specifically the clinical utility and rule-out rate of CxbM when used in routine surveillance of patients at low or high risk of recurrent bladder cancer.

## Methods

### Design

This was a retrospective audit of cystoscopy patterns and results following the introduction of CxbM into the real-world surveillance of all patients being evaluated for UC recurrence.

**Fig. 1:**
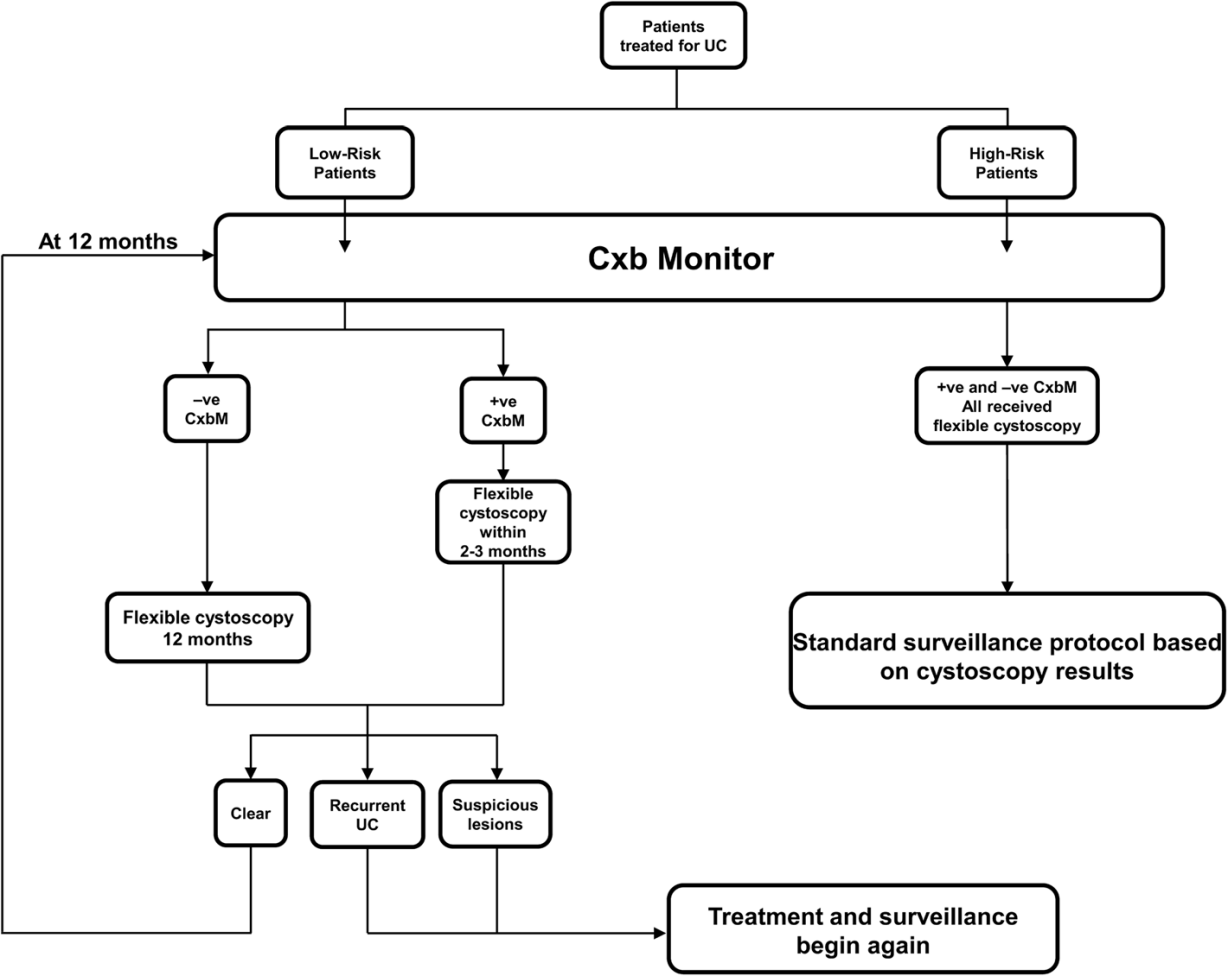
Protocol using Cxbladder-Monitor for surveillance of recurrent bladder cancer. –ve, negative; +ve, positive; CxbM, Cxbladder-Monitor; UC, urothelial carcinoma

Three PHPs in New Zealand (Waitemata, Mid-Central, Capital & Coast) implemented a new surveillance protocol. All patients (newly treated for UC and those being surveilled for recurrence) consented to a clinical work-up for identifying UC recurrence using the new protocol (Fig. 1). First, patients underwent clinical assessment of recurrence risk based on previous bladder cancer history, where patients with low-grade Ta cancers were considered to be at low risk of recurrence and patients with carcinoma in situ (Cis) or any high-grade tumor were considered to be at high risk for recurrence. All patients provided a urine sample, which was analyzed using the commercial CxbM test [16, 17]. The first CxbM result defined subsequent management of low-risk patients as follows: CxbM-negative patients were considered to be at low risk for recurrence, and underwent flexible cystoscopy only at the next scheduled assessment approximately 12 months later (unless their treating physician chose to do otherwise). Subsequent annual surveillance alternated between flexible cystoscopy and CxbM testing while test results remained negative. Low-risk patients who had a positive first CxbM result were considered to be at higher risk of having a recurrence and were scheduled for cystoscopy (Fig. 1). When a low-risk patient returned a CxbM-positive or cystoscopy-positive result, the patient was then treated and the surveillance schedule continued according to whether the patient was assessed as low or high risk (see Additional file 2).

All high-risk patients had flexible cystoscopy within the normal scheduled follow-up time in accordance with the local guidelines’ standard of care for these patients (Additional file 2). The CxbM result for high-risk patients did not affect this cystoscopy surveillance protocol (CxbM data was solely collected for retrospective analysis of patients who qualified as high-risk).

All patients who presented to the clinic for recurrence evaluation, including new patients and those who have had previous evaluations for recurrence, between 25th July 2016 and 5th July 2019 in the low-risk cohort and between 31st March 2017 and 5th July 2019 in the high-risk cohort (i.e. for the 35 months from the first CxbM test under the new protocol) were included in this audit. Data were included from all patients (regardless of risk stratification) who had undergone treatment for non-muscle invasive UC and who provided a urine sample for CxbM testing at that center.

### Outcomes

The clinical utility of CxbM was based on the number of cystoscopies avoided under the new protocol compared with the former surveillance protocol, in which all assessments were by cystoscopy. A second outcome was the number of patients who developed recurrent bladder cancer at the first post-CxbM cystoscopy and over the 35-month audit; recurrent tumors were those identified by cystoscopy and confirmed by pathology.

### Statistical analysis

Within each cohort of low- and high-risk patients, the number of recurrence events at the first cystoscopy was calculated as the number of patients with a pathology-confirmed recurrent UC diagnosis as a proportion of the total number of patients in that cohort. The timing of subsequent cystoscopies and the identification of recurrent tumors at those cystoscopies were calculated and demographic and clinical characteristics summarized using mean ± standard deviation (SD) used for continuous variables. Time between CxbM result and cystoscopy was calculated as median with 95% confidence intervals (CI), and compared between the CxbM-positive and -negative groups using log rank tests; a *p*-value of < 0.05 was considered significant.

### Ethics

The analysis used anonymized data extracted from patients’ electronic medical records and was compliant with New Zealand national privacy regulations on the use of patient data. Patients provided verbal informed consent to CxbM testing, and to the surveillance protocol, following a written disclosure and in accordance with consent procedures used for local treatment protocols, and thus with appropriate local ethics oversight (and not requiring written informed consent). The Health and Disabilities Ethics Committee (HDEC) determined that the study was undertaken to assess diagnostic quality as part of the standard of care and did not require approval.

## Results

### Patients

**Fig. 2:**
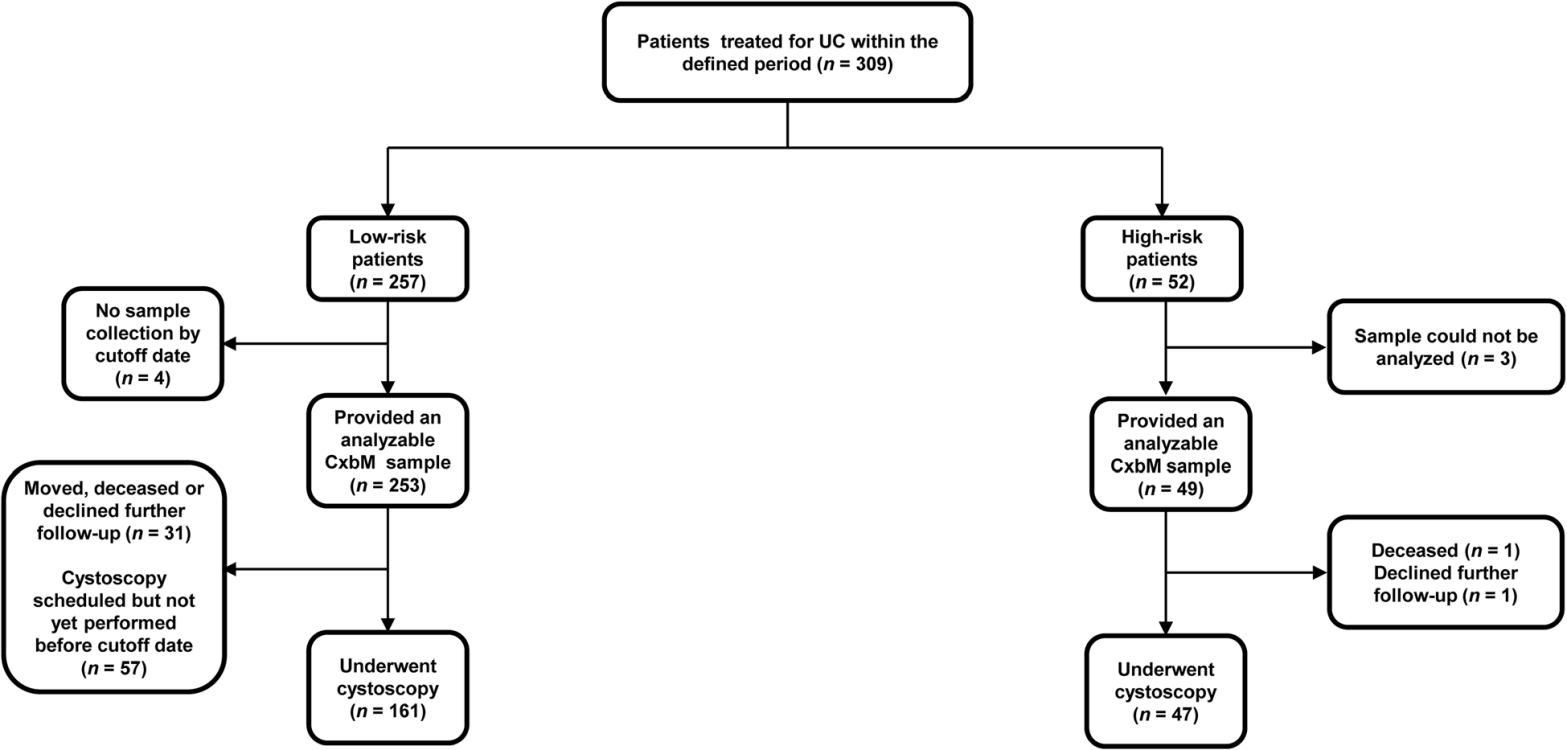
Patient flow chart for number of patients entering and completing the audit

Over the 35-month audit period, 309 patients had entered the surveillance protocol and provided 443 samples for CxbM testing. Overall, 257 (83.2%) low-risk patients provided 391 urine samples (Fig. 2), while 52 (16.8%) high-risk patients provided 52 urine samples (Fig. 2). At the time of data analysis, four low-risk patients had not provided a urine sample for CxbM, and three high-risk patients had provided a urine sample that was not available for analysis by CxbM, so 253 and 49 patients, respectively, in these groups had CxbM data available (Fig. 2). At the data cutoff, 208 patients (*n* = 161 low-risk; *n* = 47 high-risk) had undergone at least one cystoscopy.

Low-risk patients (*n* = 253) had a mean age of 73.1 years; mean time since the primary bladder cancer was 6.5 years and time since last UC treatment was 3.7 years, irrespective of this being the primary event or most recent recurrence (Additional file 3: Table S1).

### Low-risk cohort

**Fig. 3:**
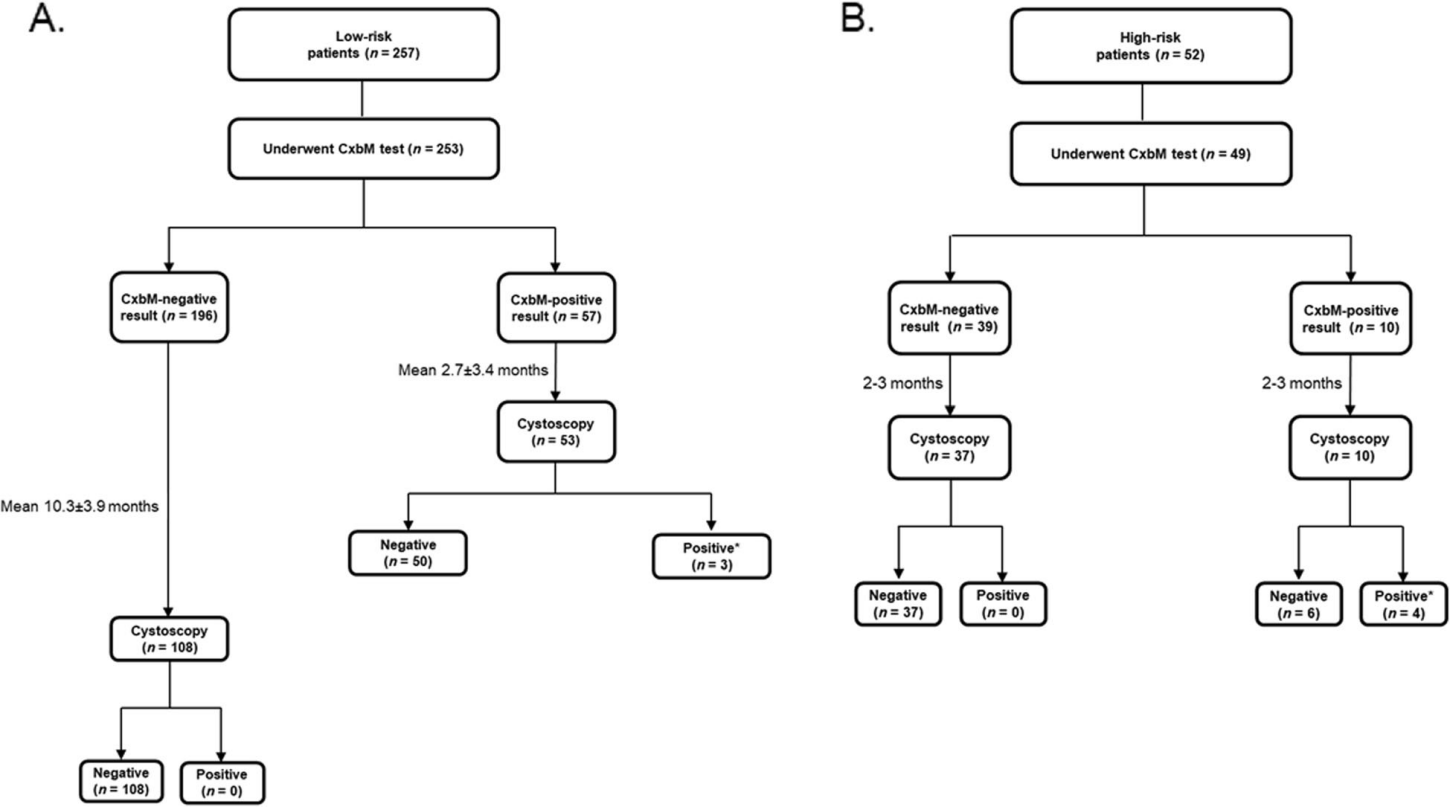
Patients completing the Cxbladder-Monitor test and flexible cystoscopy according to the protocol for (**a**) low-risk and (**b**) high-risk patients. Mean time to follow-up flexible cystoscopy is the time from the CxbM test. CxbM, Cxbladder-Monitor. *Patients proceeded to treatment for recurrent UC

Overall, 196/253 (77.5%) low-risk patients had a CxbM-negative result and 57 (22.5%) had a CxbM-positive result (Fig. 3). Of the 196 patients with a CxbM-negative result, 108 underwent the first cystoscopy a mean ± SD of 10.3 ± 3.9 months after sample collection. The other 88 patients had not undergone follow-up cystoscopy by the cutoff date for the following reasons: cystoscopy was scheduled for a future date (*n* = 60), patients had moved, died or been removed from the list of those requiring follow-up (*n* = 28). At the first cystoscopy, no patients had pathology-confirmed UC, but 10 had equivocal cystoscopic findings without pathology confirmation. Follow-up (mean 3.2 ± 1.6 months) of the 10 (9.2%) low-risk CxbM-negative patients with suspicious lesions identified pathology-confirmed low-grade recurrent Ta tumors in three patients. Therefore, over 35 months, confirmed recurrence occurred in three of the 196 low-risk patients with a CxbM-negative result (1.5%; Additional file 4: Figure S1).

Of the 57 CxbM-positive patients, 53 underwent cystoscopy a mean ± SD of 2.7 ± 3.4 months after sample collection. The other four patients had not undergone follow-up cystoscopy; reasons were: scheduled for a future date, patient declined, or unknown reason. Fifty patients had a negative cystoscopy result and three had pathology-confirmed recurrent UC (Fig. 3a); all three were low-grade Ta tumors. Therefore, three confirmed recurrence events were identified in 57 CxbM-positive low-risk patients (5.3%), or in 3/53 (5.7%) when limiting the analysis to patients who had undergone cystoscopy at 2–3 months. Another 13 patients had (unconfirmed) suspicious lesions on cystoscopy (Additional file 5: Figure S2). Follow-up of these 13 low-risk CxbM-positive patients with suspicious lesions identified pathology-confirmed recurrence in six patients (4 low-grade Ta; 1 Cis; 1 papillary neoplasm of low malignant potential). Therefore, a total of nine patients who had originally been categorized as low-risk and who tested CxbM-positive had pathology-confirmed UC recurrence (9/57; 15.8%).

### High-risk cohort

Within the evaluable cohort of 49 high-risk patients, 39 (79.6%) had a CxbM-negative result and 10 (20.4%) had a CxbM-positive result (Fig. 3b). All 10 high-risk CxbM-positive patients and 37/39 high-risk CxbM-negative patients underwent cystoscopy less than 1 month after CxbM urine sample collection. The other two CxbM-negative patients were scheduled for cystoscopy but did not have the procedure (one patient declined and one deceased).

No pathology-confirmed cases of recurrence were identified in patients with a CxbM-negative result (Additional file 5: Figure S2). In high-risk patients with a CxbM-positive result, four had recurrent tumors (40%); two were high-grade and two were low-grade UCs. No patient had Cis.

### Both risk cohorts combined

Overall, 235 patients of the 302 tested returned CxbM-negative results (77.8%). None of these 235 had pathology-confirmed recurrence, with an overall pathology-confirmed recurrence rate at first cystoscopy of nil (0%) in patients testing CxbM-negative (regardless of the initial low- or high-risk categorization). Follow-up of patients with equivocal cystoscopic findings identified three more cases of pathology-confirmed recurrence in patients with an initial CxbM-negative result (*n* = 3/253; 1.2%).

Seven pathology-confirmed cases were identified at first cystoscopy in 67 patients who tested positive for CxbM, and an additional six were identified after follow-up of suspicious cystoscopies for a total of 13 confirmed recurrence events in CxbM-positive patients (*n* = 13/67; 19.4%). Therefore, there was 16.2-fold difference in the total number of confirmed recurrences between patients with CxbM-positive and -negative results.

During 35 months of follow-up, no patients progressed to invasive disease or metastases and there was no cancer-specific mortality.

### Time since last tumor

**Fig. 4:**
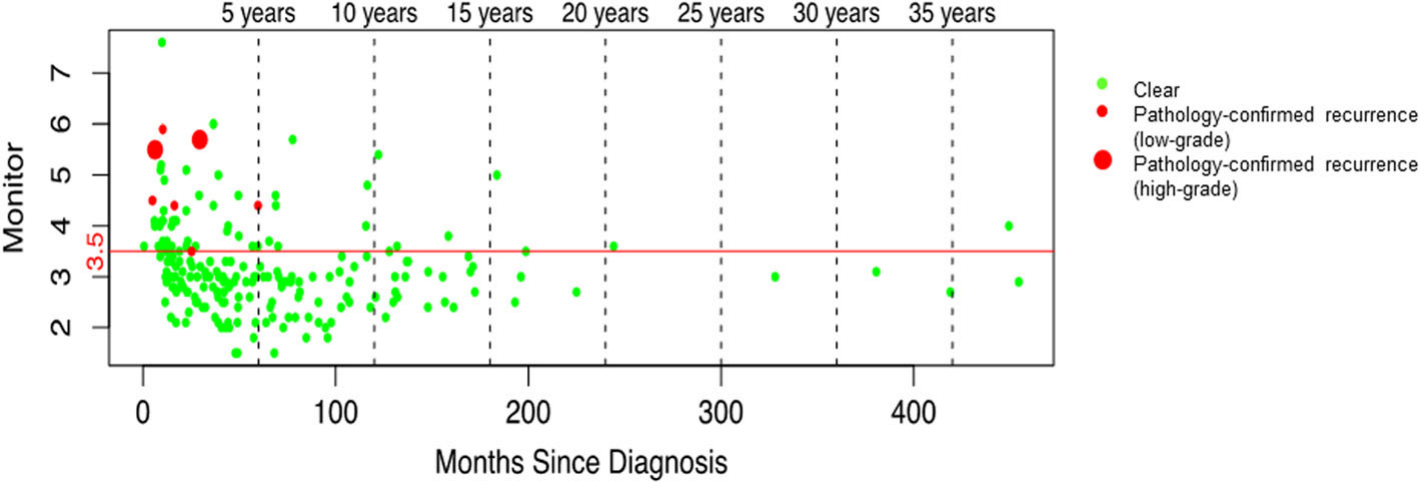
Time since last tumor recurrence in all patients with Cxbladder-Monitor-positive results. The red line indicates the CxbM score threshold for defining positive (≥3.5) and negative (< 3.5). CxbM, Cxbladder-Monitor

Figure 4 shows the time of cystoscopy relative to the time of the last tumor in all low-risk and high-risk patients, irrespective of CxbM result. CxbM identified all seven of the confirmed recurrence events identified on the first cystoscopy after CxbM introduction. Additional file 6: Figure S3 shows the same data but includes suspicious cystoscopy findings.

### Relationship between CxbM and time to cystoscopy

The median time to the first cystoscopy was 12.13 (95% CI: 11.97–12.4) months in patients with a CxbM-negative result compared with 1.63 (95% CI: 1.13–2.3) months in patients with a CxbM-positive result (*p* < 0.00001; Additional file 4: Figure S1 and Additional file 5: Figure S2).

## Discussion

This audit demonstrated the real-world clinical utility of CxbM as a rule-out test for both low- and high-risk patients undergoing surveillance for recurrent UC. The data showed no advantage to patients being segregated on the basis of risk prior to the use of CxbM.

There were no incidences of pathology-confirmed recurrence at the post CxbM cystoscopy testing ~ 10 months later. Three patients with an equivocal cystoscopy finding were pathology confirmed at a subsequent follow-up ~ 3 months later. Overall, for low-risk and high-risk patients, a CxbM-positive result was associated with a 16.2-fold greater likelihood of confirmed UC on initial cystoscopy compared with CxbM-negative findings. High-grade tumors were seen in only two patients (0.79%) who had been initially categorized as being at high risk for recurrence. One additional low-risk patient progressed to a Cis. All three had a CxbM-positive result.

Previous studies have shown that CxbM has high sensitivity and NPV [16, 17], and the current audit demonstrates that CxbM provides tangible clinical utility when used as a rule-out test to identify patients at low risk of recurrence who do not need a cystoscopy and identify those at higher risk who would benefit from cystoscopy. The integration of CxbM into local practice guidelines identified a high proportion of patients (77.8%) who were safely managed by only one cystoscopy every 2 years. Reducing by half the number of cystoscopies in this portion of patients treated for UC would decrease the total number of annual cystoscopies needed by 39%, thereby significantly reducing long-term costs of UC surveillance, without compromising detection, and enabling resources to be focused on patients most in need.

CxbM incorporates risk factors in its validated algorithm, providing an objective, repeatable measure. The audit showed that use of CxbM as a rule-out test in all recurrence patients obviates the need for risk stratification because CxbM identifies those at high risk of recurrence, irrespective of their guideline-defined risk stratification.

Other noninvasive biomarker assays have been approved in the US for the diagnosis or monitoring of bladder cancer including an Enzyme-Linked Immunosorbent Assay (ELISA) test for Nuclear Matrix Protein 22 (NMP22) (BladderChek®; Matritech Inc., Newton, MA, USA [18, 19]), a multiprobe fluorescence in situ hybridization (FISH) test (UroVysion®; Abbott Molecular, Des Plaines, IL) and an immunocytologic fluorescent assay (ImmunoCyt™/uCyt™ Diagnocure; Quebec City, Quebec, Canada). However, it has been previously observed they provide low overall sensitivity [20]. A previous study compared CxbM with NMP22 ELISA assays and UroVysion FISH in patients previously diagnosed with UC undergoing monitoring for recurrence [17]. CxbM provided significantly better sensitivity and NPV than BladderCheck (91% vs. 11% and 96% vs. 86%, respectively), and in a smaller patient sample, showed a similar advantage over UroVysion FISH (sensitivity 33%, NPV 92%) [17]. These data suggest that the NMP22 point-of-care test is likely to miss a substantial number of patients with recurrence, whereas CxbM does not.

Currently, there are limited data on the clinical value of introducing urinary biomarkers into the surveillance protocol for recurrent UC, and most studies have used the early generation, single biomarker tests with low performance [21, 22]. To our knowledge, this study is the first to have investigated the impact of incorporating a multi-biomarker urine test into a routine clinical surveillance protocol in a real-world setting. Further longer-term studies should be conducted to confirm our findings.

Collection of a urine sample carries a significantly lower burden for patients compared with cystoscopy in terms of time away from work, anxiety, pain and discomfort during the procedure, and painful micturition afterwards and is likely to lead to an increase in patient compliance with physician recommendation [11]. The results of in-office cystoscopy may be available sooner than the results of some out-sourced biomarker tests, which may limit patient anxiety compared with waiting for a result [23, 24]. However, not all cystoscopies provide a clear result, and patients may need to undergo further testing if cystoscopy is equivocal or cytology atypical [25].

Adding urine biomarker testing to a standard regimen of cystoscopy may not be cost effective when added to the standard tests and procedures for each scheduled assessment [26, 27]; however reducing the frequency of cystoscopy would significantly reduce the cost of post-treatment UC surveillance in low-risk patients [28]. The more sensitive and accurate the urine biomarker test is, the more cost-effective it is in the surveillance of recurrent UC [26], and the more acceptable it becomes to patients as an alternative to routine cystoscopy [29].

Our data have clinical implications for the surveillance of UC patients after treatment. First, because of the high sensitivity (91–95%) and NPV (96–97%) of the CxbM test, a voided urine sample can be used to rule out a substantial number of both high- and low-risk patients who are very unlikely to have recurrent UC and can safely miss one of the recommended cystoscopies, saving money and sparing patients the discomfort and anxiety. Our study also showed that CxbM effectively identified patients at higher risk of recurrence regardless of the time since the original UC diagnosis, and therefore can be implemented at any time during the post-treatment course of the disease. All three centers included in this audit now use the CxbM test in their clinical protocols to rule-out low risk patients and prioritize UC patients for follow-up cystoscopy.

This audit is not without limitations. Because this was a real-world analysis of clinical practice, a complete dataset was not available for each patient, no data were available of treatments received, and some patients were lost to follow-up through real-world events such as moving, change of contact details, or death from co-morbidities. Some patients had both CxbM and cystoscopy simultaneously, reflecting patient-specific variation in the implementation of the new standard protocols. Variability in the timing of the first post-CxbM cystoscopies and differences in data availability existed between the low- and high-risk groups (e.g. 391 urine samples vs. 52 urine samples, respectively), and some low-risk patients had more than one cystoscopy and recurrence, which impacted on the comparison of recurrence rates between the CxbM-positive and -negative patient groups over time. The difference in the number of urine samples between the low-risk and high-risk patients was partly an artefact of the new surveillance protocol, where high-risk patients underwent frequent surveillance by cystoscopy, rather than by CxbM, whereas low-risk patients had an alternating surveillance regimen (CxbM then cystoscopy, then CxbM and so on). Finally, low-risk CxbM-negative patients did not undergo any conventional follow-up until the 12-month cystoscopy, so we are unable to confirm whether the 12-month equivocal cystoscopy events were developed after CxbM testing. Any comparison between CxbM and cystoscopy results in the low-risk patient group is similarly limited by missing data, as a consequence of adopting this alternating CxbM to cystoscopy protocol.

Strengths of this study are that we included a large sample of patient data collected over a 35-month audit period, during real-world clinical practice, in which clinical decisions were made based on the results of the CxbM molecular test.

## Conclusion

When integrated into the evaluation of all patients under surveillance for bladder cancer recurrence, CxbM accurately ruled out patients who did not have recurrent UC. This enabled all low-risk patients to safely undergo cystoscopy at a longer-than-recommended interval, thereby reducing the cystoscopy burden, and sparing patients the potential discomfort and anxiety associated with the procedure. CxbM missed no high grade tumors or failed to identify high-risk patients and the rate of pathology-confirmed UC recurrence was 16.2-fold lower in CxbM-negative than CxbM-positive patients. The current study results indicate that CxbM provides an objective and repeatable measure of recurrence, obviating the need for risk stratification based on clinical parameters.

## Supplementary information

**Additional file 1.** Cxbladder Monitor description and validation results.

**Additional file 2.** Flexible cystoscopy surveillance protocols.

**Additional file 3: Table S1.** Patient demographic and clinical characteristics.

**Additional file 4: Figure S1.** Time from initial Cxbladder-Monitor (CxbM) test to cystoscopy in all low-risk patients anchored on first CxbM result.

**Additional file 5: Figure S2.** Time from initial Cxbladder-Monitor (CxbM) test to cystoscopy in all high-risk patients anchored on first CxbM result.

**Additional file 6: Figure S3.** Time from last tumor recurrence to the first cystoscopy after Cxbladder Monitor (CxbM) introduction in all patients with CxbM results. The red line indicates the CxbM score threshold for defining positive (≥3.5) and negative (< 3.5). CxbM, Cxbladder-Monitor.

## Data Availability

All available data are included in this manuscript.

## Abbreviations

AUA: American Urological Association
CI: Confidence interval
Cis: Carcinoma in situ
CxbM: Cxbladder Monitor
ELISA: Enzyme-linked immunosorbent assay
FISH: Fluorescence in situ hybridization
HDEC: Health and Disabilities Ethics Committee
NMP22: Nuclear Matrix Protein 22
NPV: Negative predictive value
PHP: Public healthcare provider
SD: Standard deviation
UC: Urothelial carcinoma
