# Supplementary material for: An evaluation of the real world use and clinical utility of the Cxbladder Monitor assay in the follow-up of patients previously treated for bladder cancer
Source: BMC Urol. 2020 Feb 11;20:12. doi: 10.1186/s12894-020-0583-0 (PMC7014779; doi:10.1186/s12894-020-0583-0)
Supplement: Supplementary file 1 — Additional file 1. Cxbladder Monitor description and validation results. [file 12894_2020_583_MOESM1_ESM.docx]

# Additional file 1

## Cxbladder Monitor description and validation results

Cxbladder Monitor (CxbM) was designed to rule out the presence of recurrent urothelial carcinoma (UC) from among those who have previously received treatment for UC. From a single 5 ml sample of urine, this test quantifies urine mRNA levels of the following five cancer biomarkers: *IGFBP5*, *HOXA13*, *MDK*, *CDK1* and *CXCR2* [1]. This information is combined with clinical variables into a mathematical algorithm to derive a score that provides a binary outcome [1].

High sensitivity and high negative predictive value were confirmed in prospective studies in patients undergoing surveillance for recurrent bladder cancer [1, 2]. The validation cohort consisted of 1036 urine samples collected prospectively from 763 patients undergoing routine surveillance for recurrent UC of the bladder [1]. Cxbladder Monitor had an internally validated sensitivity of 0.93 with a negative predictive value of 0.97 and a test-negative rate of 0.34 [1]. Sensitivity was 0.95 for recurrent disease with a high risk of progression (i.e. all high-grade disease and low-grade disease at stage ≥T1) and 0.86 for low-grade Ta disease. Diagnostic performance was independent of patient age, gender, tumor stage and adjuvant Bacillus Calmette-Guérin treatment in the prior 6 months, according to subgroup analyses [1]. False-negatives were reported in <1.5% of all samples collected.

References

1. Kavalieris L, O'Sullivan P, Frampton C, et al. Performance Characteristics of a Multigene Urine Biomarker Test for Monitoring for Recurrent Urothelial Carcinoma in a Multicenter Study. J Urol. 2017;197(6):1419-26.

2. Lotan Y, O'Sullivan P, Raman JD, et al. Clinical comparison of noninvasive urine tests for ruling out recurrent urothelial carcinoma. Urol Oncol. 2017;35(8):531 e15- e22.
