# Supplementary material for: An evaluation of the real world use and clinical utility of the Cxbladder Monitor assay in the follow-up of patients previously treated for bladder cancer
Source: BMC Urol. 2020 Feb 11;20:12. doi: 10.1186/s12894-020-0583-0 (PMC7014779; doi:10.1186/s12894-020-0583-0)
Supplement: Supplementary file 2 — Additional file 2. Flexible cystoscopy surveillance protocols. [file 12894_2020_583_MOESM2_ESM.docx]

**Additional file 2**

## Flexible cystoscopy surveillance protocol

**High-risk patients**

Local guidelines in use at the three participating public healthcare providers (PHP) describe the standard surveillance protocol for monitoring high-risk patients. Briefly, the protocol is as follows:

1. Patients identified as high-risk for recurrence of disease are monitored for recurrence of disease by scheduled cystoscopy every 3 months for the first 6 months following resection and if the patient remains clear of disease, then every 6 months thereafter. Sampling for the CxbM test was conducted prior to the scheduled visit for cystoscopy.
2. Patients with a non-clear cystoscopy are treated (tumor resection), and surveillance for disease recurrence recommences on a schedule as per the above.

**Low-risk patients**

The standard surveillance protocol in the local guidelines for low-risk patients is flexible cystoscopy at 4 months following tumor resection, and if clear, annually thereafter.
