# Supplementary material for: An evaluation of the real world use and clinical utility of the Cxbladder Monitor assay in the follow-up of patients previously treated for bladder cancer
Source: BMC Urol. 2020 Feb 11;20:12. doi: 10.1186/s12894-020-0583-0 (PMC7014779; doi:10.1186/s12894-020-0583-0)
Supplement: Supplementary file 3 — Additional file 3: Table S1. Patient demographic and clinical characteristics. [file 12894_2020_583_MOESM3_ESM.docx]

Additional file 3: **Table S1.** Patient demographic and clinical characteristics

|  | **Low-risk patients (*n* = 253)** | **High-risk patients (*n* = 50)** |
| --- | --- | --- |
| Mean age, years | 73.1 | N/A |
| Gender, n (%) |  |  |
| Male | 109^a^ (68) | N/A |
| Female | 52^a^ (32) | N/A |
| Data unavailable | 92 | N/A |
| Mean time since most recent UC treatment^b^, years | 3.7 | N/A |
| Mean time since primary cancer, years | 6.5 | 4.5 |

# ^a^Denominator is 161 patients with gender data available

^b^For primary or recurrent UC.

# N/A, not available
